# Evolutionary concepts can benefit both fundamental research and applied research in toxicology (A comment on Brady et al. 2017)

**DOI:** 10.1111/eva.12695

**Published:** 2018-11-05

**Authors:** Mark E. Hahn

**Affiliations:** ^1^ Biology Department Woods Hole Oceanographic Institution Woods Hole Massachusetts; ^2^ Boston University Superfund Research Program Boston University School of Public Health Boston Massachusetts; ^3^ Woods Hole Center for Oceans and Human Health Woods Hole Massachusetts

In their insightful overview of the special issue of *Evolutionary Applications* on Evolutionary Toxicology, Brady, Monosson, Matson, and Bickham (2017) provide a much‐needed call for the application of evolutionary concepts in our efforts to understand life's responses to toxic chemicals. I write to comment on one aspect of their editorial that deserves a broader perspective.

Brady and coauthors refer several times to toxicology as an “applied” science. Indeed, there are important applications of toxicology, for example, in toxicity testing of chemicals or in human health and ecological risk assessment. However, I would argue that toxicology is much more than an applied science.

Important toxicological research in the past and much of the toxicological research occurring today should be considered basic or fundamental research, rather than applied. For example, toxic chemicals—including both natural products such as tetrodotoxin and synthetic chemicals like dioxin (2,3,7,8‐tetrachlorodibenzo‐*p*‐dioxin)—have long been used as “molecular probes” to investigate fundamental aspects of cell and molecular biology (Narahashi, 1977; Poland & Kende, 1976). Research in toxicology (and its sibling, pharmacology) has provided fundamental insights into the biochemistry of enzymes that catalyze the biotransformation of both xenobiotic and endogenous chemicals (Lu, 1998; Nebert & Gonzalez, 1987; Nelson, Goldstone, & Stegeman, 2013). Transcription factors discovered because of their roles in the response to chemicals have subsequently been found to have fundamental roles in development, physiology, and immunology (Esser & Rannug, 2015; Nebert, 2017; Oladimeji & Chen, 2018; Sykiotis & Bohmann, 2010).

Even much of the toxicological research performed in support of applied goals such as testing or risk assessment is of a fundamental nature. Many examples can be found in the extensive research on mechanisms of toxicity, which generates basic understanding that informs screening efforts (Martin et al., 2010; Sipes et al., 2013) and regulatory decision‐making (Clewell, 2005; Haber et al., 2001; Sturla et al., 2014). Such research might best be considered fundamental research inspired by societal needs or “use‐inspired basic research” as defined by Stokes (1995, 1997).

The concept of *Evolutionary Toxicology* encompasses at least two distinct but related ideas, both of which are noted in Brady et al. (2017). The first, as outlined in the foundational description of *Evolutionary Toxicology* (Bickham & Smolen, 1994), concerns how exposure to chemicals can, by causing mutations or imposing strong selective pressures, drive the evolution of populations and species (Bickham, 2011; Bickham, Sandhu, Hebert, Chikhi, & Athwal, 2000; Di Giulio & Clark, 2015; Klerks, Xie, & Levinton, 2011; Nacci, Champlin, & Jayaraman, 2010; Oziolor, Bickham, & Matson, 2017; Oziolor & Matson, 2015; Reid et al., 2016). The second involves understanding how deep evolutionary history has shaped animal responses to chemicals, including mechanisms of toxicity (Ballatori, Boyer, & Rockett, 2003; Ballatori & Villalobos, 2002) and defense (Goldstone et al., 2006; Nebert & Dieter, 2000) and using that information to inform both basic research and applied research in toxicology. For example, understanding the evolutionary basis of phenotypic plasticity during development provides insight into fundamental mechanisms underlying the developmental origins of adult disease (Gluckman, Hanson, & Beedle, 2007; Lea, Tung, Archie, & Alberts, 2017). Such an evolutionary perspective, which has parallels in the emerging field of *Evolutionary Medicine* (Nesse & Stearns, 2008; Stearns, 2012; Stearns, Nesse, Govindaraju, & Ellison, 2010; Wells, Nesse, Sear, Johnstone, & Stearns, 2017), can guide the selection of model systems in toxicological research and inform the extrapolation of results from those models to humans or wildlife (e.g., Gunnarsson, Jauhiainen, Kristiansson, Nerman, & Larsson, 2008; Lalone et al., 2013; Leung et al., 2017).

The thesis of Brady et al. (2017)—that an evolutionary perspective can benefit toxicology—is one with which I strongly agree (Hahn, 2002; Hahn, Karchner, & Merson, 2017; Whitehead, Clark, Reid, Hahn, & Nacci, 2017). However, evolutionary concepts can enrich more than just the applied forms of toxicology; they also provide an important framework that enhances the fundamental understanding of toxicological mechanisms and the basic biology of the genes and proteins that control life's response to toxic chemicals.
