# Supplementary material for: Evolutionary concepts can benefit both fundamental research and applied research in toxicology (A comment on Brady et al. 2017)
Source: Evol Appl. 2018 Nov 5;12(2):350–2. doi: 10.1111/eva.12695 (PMC6346646; doi:10.1111/eva.12695)
Supplement: Supplementary file 1 [file EVA-12-350-s001.docx]

**Fundamental and applied pursuits in evolutionary toxicology are mutually beneficial: A reply to Hahn (2018)**

Hahn (2018) asserts that evolutionary concepts can benefit not only applied research, but also fundamental research in toxicology. We could not agree more. While our introduction to the recent special issue on Evolutionary Toxicology mentioned the reciprocal value toxicology offers basic evolutionary research (Brady, Monosson, Matson, & Bickham, 2017), we agree that our treatment of evolutionary toxicology focused largely on applied contexts. Yet as Hahn accurately describes, toxicology is much more than an applied science, and the infusion of evolutionary concepts benefits many lines of fundamental toxicology research. Indeed, this point resonates with previous perspectives showcasing the value of evolutionary concepts to advance basic toxicology (e.g., Monosson, 2012), such as insights into cytochrome P450 evolution and corresponding enzyme function across taxa (Sezutsu, Le Goff, & Feyereisen, 2013).

It is worth considering how our own backgrounds in ecological research might have biased our attention away from fundamental aspects of evolutionary toxicology. For many who study wild populations, there is a sense of obligation to suggest applications for research findings, even if those suggestions might be out of touch with practice. This premium for applied relevance can neglect the critical role and value of scientific curiosity and fundamental research (Courchamp et al., 2015). It is perhaps with a similar sense of obligation that we focused on applied aspects of toxicology despite its fundamental research value. We are therefore grateful for Hahn’s reminder of the vital importance of fundamental research.

Ultimately, the most comprehensive understanding in toxicology might best be achieved through dual pursuits of both basic and applied research. As Hahn notes, even applied toxicology is fundamental in nature. We suggest further that applied insights can help inform basic insights. For instance, novel conditions or objectives in applied contexts might help elucidate novel mechanisms underlying toxicological responses. Thus, the relationship between applied and basic research in toxicology is one of mutual benefit, each inspiring the other and leading to advancement of knowledge.

We appreciate the important and balanced perspective put forth by Hahn, and are encouraged that our special issue on Evolutionary Toxicology is prompting consideration of the diverse value of the role of evolutionary concepts in toxicology. We look forward to future discoveries and syntheses that await this vibrant and multidisciplinary field.

**Literature Cited**

Brady, S. P., Monosson, E., Matson, C. W., & Bickham, J. W. (2017). Evolutionary toxicology: Toward a unified understanding of life's response to toxic chemicals. *Evolutionary Applications, 10*(8), 745-751. doi:10.1111/eva.12519

Courchamp, F., Dunne, J. A., Le Maho, Y., May, R. M., Thébaud, C., & Hochberg, M. E. (2015). Fundamental ecology is fundamental. *Trends in Ecology & Evolution, 30*(1), 9-16.

Hahn, M. E. (2018). Toxicology is more than an applied science: Evolutionary concepts can benefit both applied and fundamental research in toxicology (A comment on Brady et al. 2017). *Evolutionary Applications*.

Monosson, E. (2012). *Evolution in a Toxic World: How Life Responds to Chemical Threats*. Washington, DC: Island Press.

Sezutsu, H., Le Goff, G., & Feyereisen, R. (2013). Origins of P450 diversity. *Phil. Trans. R. Soc. B, 368*(1612), 20120428.
